# Genetic diversity of *Plasmodium falciparum* and distribution of drug resistance haplotypes in Yemen

**DOI:** 10.1186/1475-2875-12-244

**Published:** 2013-07-15

**Authors:** Salama Al-Hamidhi, Mohammed AK Mahdy, Zainab Al-Hashami, Hissa Al-Farsi, Abdulsalam M Al-mekhlafi, Mohamed A Idris, Albano Beja-Pereira, Hamza A Babiker

**Affiliations:** 1 Department of Biochemistry, Faculty of Medicine and Health Sciences, Sultan Qaboos University, Alkhod, PO Box 35, Muscat, Oman; 2 Department of Microbiology and Immunology, Faculty of Medicine and Health Sciences, Sultan Qaboos University, Muscat, Oman; 3 Department of Parasitology, Faculty of Medicine, University of Malaya, Kuala Lumpur, Malaysia; 4 Department of Parasitology, Faculty of Medicine, Sana’a University, Sana’a, Yemen; 5 Research Centre in Biodiversity and Genetic Resources (CIBIO), University of Porto, Rua Padre Armando Quintas 7, Vairão, Portugal; 6 Institute of Immunology and Infection Research, School of Biological Sciences, University of Edinburgh, Edinburgh EH9 3JT, UK

**Keywords:** Malaria, Yemen, Arabian Peninsula, Drug resistance, Plasmodium falciparum, Genetic diversity

## Abstract

**Background:**

Despite evident success of malaria control in many sites in the Arabian Peninsula, malaria remains endemic in a few spots, in Yemen and south-west of Saudi Arabia. In addition to local transmission, imported malaria sustains an extra source of parasites that can challenge the strengths of local control strategies. This study examined the genetic diversity of *Plasmodium falciparum* in Yemen and mutations of drug resistant genes, to elucidate parasite structure and distribution of drug resistance genotypes in the region.

**Methods:**

Five polymorphic loci (*MSP-2*, *Pfg377* and three microsatellites on chromosome 8) not involved in anti-malarial drug resistance, and four drug resistant genes (*pfcrt*, *pfmdr1*, *dhfr* and *dhps*) were genotyped in 108 *P*. *falciparum* isolates collected in three sites in Yemen: Dhamar, Hodeidah and Taiz.

**Results:**

High diversity was seen in non-drug genes, *pfg377* (*He* = 0.66), *msp*-*2* (*He* = 0.80) and three microsatellites on chr 8, 7.7 kb (*He* = 0.88), 4.3 kb (*He* = 0.77) and 0.8 kb (*He* = 0.71). There was a high level of mixed-genotype infections (57%), with an average 1.8 genotypes per patient. No linkage disequilibrium was seen between drug resistant genes and the non-drug markers (p < 0.05). Genetic differentiation between populations was low (most pair-wise *F*_ST_ values <0.03), indicating extensive gene flow between the parasites in the three sites.

There was a high prevalence of mutations in *pfmdr1*, *pfcrt* and *dhfr*; with four mutant *pfmdr1* genotypes (NFCDD[57%], NFSND[21%], YFCDD[13%] and YFSND[8% ]), two mutant *pfcrt* genotypes (CVIET[89%] and SVMNT[4%]) and one mutant *dhfr* genotype (ICNI[53.7%]). However, no *dhps* mutations were detected.

**Conclusion:**

The high diversity of *P*. *falciparum* in Yemen is indicative of a large parasite reservoir, which represents a challenge to control efforts. The presence of two distinct *pfcrt* genotype, CVIET and SVMNT, suggests that chloroquine resistance can possibly be related to a migratory path from Africa and Asia. The absence of the triple mutant *dhfr* genotype (IRN) and *dhps* mutations supports the use of artesunate + sulphadoxine-pyrimethamine as first-line therapy. However, the prevalent *pfmdr1* genotype NFSND [21%] has previously been associated with tolerance/resistance response to artemisinin combination therapy (ACT). Regular surveys are, therefore, important to monitor spread of pfmdr1 and dhfr mutations and response to ACT.

## Background

The Arabian Peninsula lies at the fringes of malaria endemicity where successful control measures have brought local transmission to halt in many countries in the region, e.g. Bahrain, Kuwait and United Arab Emirates (UAE) [1]. However, some sites in Yemen and southern Saudi Arabia remain malarious, with a high prevalence of drug-resistant *Plasmodium falciparum* parasites [2,3]. In addition, imported malaria cases, via asymptomatic travellers from malaria-endemic areas, sustain a major challenge for possible rebound of local transmission [4].

The region attracts numerous migrations from Africa and Asia. Over the past two decades political instability has resulted in mass movement of people from the Horne of Africa (Somalia, Eritrea and Ethiopia) into Yemen and Saudi Arabia [5]. These countries are known for high prevalence of drug-resistant *P. falciparum* malaria. In addition, the region attracts a lot of skilled workers from Asia. However, the impact of these migrations on the gene pool of local malaria parasites in the Arabian Peninsula remains unclear as information on malaria parasites genetics is scarce.

In Yemen, over 60% of the population live in areas with stable malaria transmission [6]. *Plasmodium falciparum* is responsible for about 90% of malaria cases. Indigenous chloroquine resistance (CQR) was reported in 1989 [7,8], and since then it spread to different regions in the highland and lowland areas in Taiz [7] and Hodeidah [9]. Cross- sectional surveys in 2008 and 2009 in Taiz, Hodeidah, Dhamar and Rymah showed high prevalence of the CQR marker *pfcrt* 76 T [2]. However, resistance to sulphadoxine-pyrimethamine (SP) is rare in Yemen [2,10]. Currently SP is used in combination with artesunate (AS) as first-line treatment of uncomplicated falciparum malaria. In addition, artemether-lumefantrine (AL) is used as a second-line therapy for treatment failure [11].

The present study examined genetic diversity of *P. falciparum* in Yemen, and distribution of genes implicated in resistance to widely used anti-malarial drugs, including, chloroquine, SP, mefloquine and artemisinin. Such analysis will provide desirable information on the genetics structure of *P. falciparum* in the Arabian Peninsula, and the prospect of evolution of drug resistance following recent shift of malaria therapy to artemisinin combination therapy (ACT).

## Methods

### Study sites and sampling

**Figure 1:**
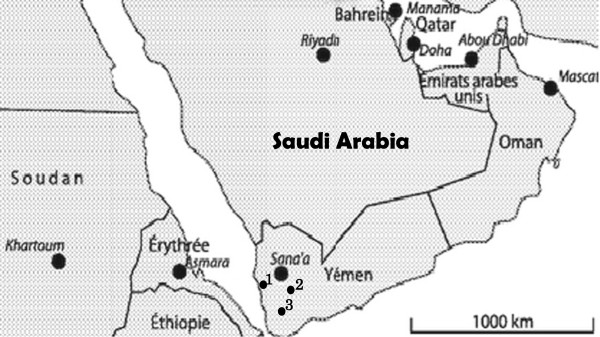
**Geographical location of the three studied sites in Yemen**: **Hodeidah (1), Dhamar (2), and Taiz (3).**

One hundred and fifteen finger-prick blood samples were collected, from microscopy-confirmed malaria cases, in three localities in Yemen (Dhamar, Hodeidah and Taiz) (Figure 1) Dhamar (Latitude: 14°32′33″ N, Longitude: 44°24′18″ E ) is a highland (2407 m above sea) where transmission occurs throughout the year. Hodeidah (Latitude: 14°48′08″N, Longitude: 42°57′04″E) is a coastal area where malaria transmission occurs in winter and Taiz (Latitude: 13°34′44″N, Longitude: 44°01′19″E) is a mountainous area (1311 m above sea) and transmission occurs in summer [2]. Ethical clearance was obtained from Faculty of Medicine, Sana’a University, Yemen. Informed consent was obtained from each participant before enrolment in the study.

### Non drug-resistance genes

#### *pfg377* and *msp-2*

The *pfg377* gene primers amplify a polymorphic region, encoding a seven degenerate amino acid repeats; alleles of this gene, vary by multiples of 21 base pairs [12]. Polymerase chain reaction (PCR) primers and conditions for amplification were as described previously [12], with minor modification where the nested primer 377R3D1 was labelled.

*msp-2* alleles fall into two sequence types, FC27 and 3D7, which differ in the sequence of the dimorphic central region, block 3 [13]. PCR primers and conditions for amplification of each type were described by Aubouy *et al.*[14].

The fluorescent PCR products of *pfg377* and *msp-2* were analysed in an ABI 3130XL Genetic Analyzer, and alleles were visualized and sized on Genesmapper v. 4 (Applied Biosystems).

### Microsatellites

Three single copy microsatellites on chr 8 flanking the *dhps* (0.8, 4.3 and 7.7 kb from the 3′ end of the gene) were types. All isolates examined in this study harbour the wild-type *dhps*, therefore, the above microsatellites are not influenced by drug pressure and were used to examine parasite diversity and population structure.

The microsatellites were examined as described by Roper *et al.*[15]. Fluorescent PCR products were analysed in an ABI 3130XL Genetic Analyzer, and alleles were visualized and sized on Genemapper v.4 (Applied Biosystems).

### Drug resistance genes

#### *pfcrt*, *pfmdr1, dhfr* and *dhps*

Alleles of the *pfcrt*, *pfmdr1*, *dhfr* and *dhps* genes were amplified using two rounds of PCR [1,16-19]. The amplified fragments of each gene encompasses mutations associated with CQR (*pfcrt*-72,-74,-75, and −76), and multidrug resistant gene-1 (*pfmdr1*-86, -184, 1034,1042 and 1246), pyrimethamine resistance (*dhfr*-51, -59, -108, and −164), sulphadoxine resistance (*dhps*-436, -437, -540, and −613).

PCR fragments of the isolates, together with control clones (3D7, Hb3 and Dd2) of known sequence and drug response, were then sequenced. The parasites were obtained from The European Malaria Reference Reagent Repository. 3D7 and Dd2, are chloroquine sensitive and resistant clones, with pfcrt76K and pfcrt76T codons, respectively [20], and codons *pfmdr1* 86 N and 86Y associated with susceptible and resistance response to choloroquine, respectively [21]. The 3D7 clone is known to be pyrimethamine and sulphadoxine sensitive carrying wild-type *dhfr* and *dhps* gene, implicated in resistance to the two drug respectively [22], while Dd2 is a pyrimethamine and sulphadoxine resistance, carrying mutations in *dhfr* and *dhps* associated with in vitro resistance [23]. The PCR products were first purified using ExoSAP-IT (USB) as described by the manufacturer. Sequencing was carried out using BigDye Terminator v3.1 cycle (ABI, UK) and run on a thermocycler. Initial sequences were compared with reference sequences in the PlasmoDB database of *P. falciparum* genomic using BLASTN, BioEdit and Lasergene software.

### Statistical analysis

The prevalence of an allele and genotype were calculated as percentage of all the alleles and genotypes detected at a given locus among the examined isolates. The expected heterozygosity index (*H*e), which measures the diversity at a locus examined, was calculated for non-drug resistant genes using microsatellite Toolkit software as described elsewhere [24].

The multiplicity of infection was defined as the proportion of people who carry more than one allele (genotype) for any of the examined genes, and the minimum number of clones per infection was estimated as the largest number of alleles at any of the examined loci [25].

Gamete linkage disequilibrium (LD) values between all pairs of loci were calculated for all samples GENEPOP v.4.1 [26]. For these analyses, only the predominant alleles at each locus, indicating clonal infections, were used.

To examine whether allele frequencies differ between parasite populations in Dhamar, Hodeidah and Taiz, *F*_*ST*_ indices were calculated using Weir and Cockerham's method [27] estimator of Wright's F-statistics using the computer package GENEPOP v.4.1 [26]. A permutation test (n = 10,000) was applied (permuting alleles over populations) to test whether *F*_*ST*_ indices were significantly different from zero.

Principal coordinate analysis (PCoA) was carried out to examine the genetic similarities (of alleles of non-drug resistant genes) between *P. falciparum* isolates in the three coding sites using Genalex Version 6.5 [28]. The two-dimensional PCoA plot shows the relationships between the haplotypic variants found in the three populations.

## Results

Out of the 115 samples with microscopy-confirmed malaria parasites, 108 proved to be *P. falciparum* and produced the expected PCR amplicons for the examined genes (*pfcrt, pfmdr1*, *dhfr*, *dhps*, *pfg377* and *msp-2*). The other seven (6.4%) isolates were most likely to have been *Plasmodium vivax*, which is found in Yemen.

### Diversity and structure of non drug-resistant genes

The five non drug-resistant loci (*pfg377*, *msp-2* and three microsatellites on chr 8) were highly polymorphic, with a number of alleles per locus ranging from five (*pfg377)* to 45 (*msp-2*). Diversity, expressed as expected hetrozygosity (*He*), was high for all loci, ranging from 0.51 to 0.89, with average of 0.66 (for *pfg377*) and 0.80 (for *msp-2*) (Table 1), reflecting high diversity among the examined isolates.

**Table 1:** **Allelic diversity (heterozygosity, *He*) at five non drug resistance loci among *Plasmodium falciparum *in three sites in Yemen**

	**Dhamar**	**Hodeidah**	**Taiz**	***F***_***st***_
	***He***	***He***	***He***	
	**(n = 29)**	**(n = 17)**	**(n = 62)**	
Chr 8				
7.7Kb	0.88	0.08	0.87	0.021
4.3Kb	0.77	0.8	0.84	0.020
0.8Kb	0.71	0.77	0.66	0.024
Chr 2				
*MSP-2*	0.61	0.51	0.89	0.141
Chr 12				
*Pfg377*	0.63	0.73	0.66	0.003
Mean	0.72	0.72	0.78	

No LD was seen between genes involved in drug resistance (*pfcrt*, *pfmdr1* and *dhfr*) and non-drug loci (*pfg377* and *msp-2*) (P < 0.001). However, loci involving resistance to one drug (*pfcrt* and *pfmdr1*) had a greater average LD than those not involved in drug resistance loci (P < 0.01).

**Figure 2:**
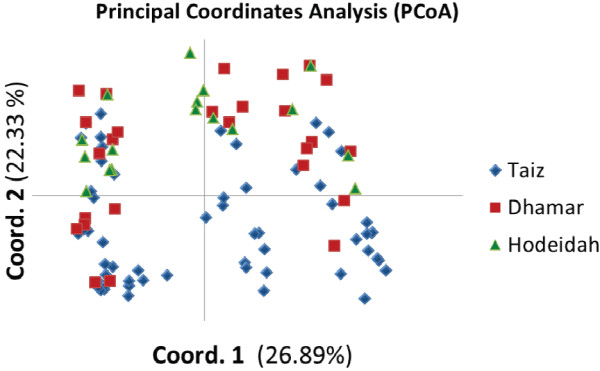
**Graphical plotting of the PCoA based on genetic variation of non drug-resistant genes (*Pfg377*, *MSP-2 *and three microsatellites in chr 8) among *Plasmodium falciparum *parasites in three sites in Yemen, and variation representation by each axis.** Blue icon = Taiz, red icon = Dhamar and green icon = Hodeidah.

Alleles at non drug-resistance loci were distributed widely across parasites in different sites in Yemen (Table 1), and any ‘private’ alleles (detected in only one population) existed at very low frequencies (Additional file 1). Comparison of parasites in the three sites showed low *F*_*st*_ values, ranged from 0.141 to 0.003, suggesting frequent gene flow (Table 1). Furthermore, PCoA analysis showed no pattern of clustering of parasites in any of the three geographical sites (Figure 2).

### Multiplicity of *Plasmodium falciparum* infection

There was a high prevalence of mixed genotype infection, 57% of the isolates carried more than one genotype. The number of genotypes per infected person (the maximum number of alleles observed at any locus) was estimated as 1.8, assuming that each clone is readily transmissible to mosquito, this reflects a high rate of cross-mating [25].

### Distribution of drug resistance markers

#### *pfcrt*

Out of the 108 *P. falciparum* isolates successfully examined, four (4%), 96 (89%), 96 (89%) and 100 (93%) harboured mutations at codons 72, 74, 75 and 76 respectively (Table 2). Ninety-six (89%), four (4%) and eight (7%) isolates carried the *pfcrt* genotypes CV**IET**, **S**VMN**T** and the wild-type (CVMNK), respectively (Table 2).

**Table 2:** **Prevalence of mutant alleles of *pfcrt, pfmdr1*, *dhfr *and *dhps *genes among 108 *Plasmodium falciparum*** in three sites in Yemen

	**All**	**Taiz**	**Dhamar**	**Hodeidah**
***pfcrt***	**n (%)**	**n (%)**	**n (%)**	**n (%)**
Alleles				
72**S**	4 (4)	0	0	4 (4)
74**I**	96 (89)	55(50.9)	29(26.9)	12(11)
75**E**	96 (89)	55(50.9)	29(26.9)	12(11)
76**T**	100 (93)	55(50.9)	29(26.9)	16(14.8)
Genotypes				
CVMNK	8 (7)	7(6.5)	0	1(1)
**S**VMN**T**	4 (4)	0	0	4 (4)
CV**IET**	96 (89)	55(50.9)	29(26.9)	12(11)
***pfmdr1***				
Alleles				
86**Y**	22 (20)	18(16.7)	3(3)	1(1)
184 **F**	107 (99)	62(57)	28(25.9)	17(15.7)
1034**C**	76 (70)	44(40.7)	20(18.5)	12(11)
1042**D**	76 (70)	44(40.7)	20(18.5)	12(11)
1246**Y**	0	0	0	0
Genotypes				
NYSND	1 (1)	0	1(1)	0
N**F**SND	23 (21)	12(11)	6(5.6)	5(4.6)
**YF**SND	8 (8)	6(5.6)	2(2)	0
N**FCD**D	62 (57)	32(29.6)	19(17.6)	11(10)
**YFCD**D	14 (13)	12(11)	1(1)	1(1)
***dhfr***				
Alleles				
51**I**	58 (54)	40(37)	12(11)	6(5.6)
59**R**	0	0	0	0
108 **N**	58 (54)	40(37)	12(11)	6(5.6)
164 **L**	0	0	0	0
Genotypes				
NCSI	50 (46)	22(20.4)	17	11(10)
**I**C**N**I	58 (54)	40(37)	12(11)	6(5.6)
***dhps***				
Alleles				
436 **F**	0	0	0	0
437**G**	0	0	0	0
540**E**	0	0	0	0
613**S**	0	0	0	0
Genotypes				
SAKA	108 (100)	62 (57)	29(26.9)	17(15.7)

n = number of isolates, mutated amino acids are bold and underlined.

All isolates carrying the wild-type *pfcrt* genotype (CVMNK) were detected in Taiz, except one seen in Hodeidah. Similarly, isolates with the mutant *pfcrt* genotype **S**VMN**T** were only seen in Hodeidah.

#### *pfmdr1*

There was a, high prevalence of *pfmdr1* mutation at codon N86**Y** (20%), Y184**F** (99%), S1034**C** (70%) and N1042**D** (70%), while no mutation was seen at codon 1246 (Table 2). The majority of isolates (57%) carried the mutant genotype (N**FCD**D), while 21%, 13%, and 8% of them harboured the mutant genotype (N**F**SND), (**YFCD**D), and (**YF**SND) respectively. However, only one isolate carried the wild-type (1%) (Table 2).

#### *dhfr*

No mutations were seen at codons 59 and 164. However, 58 (54%) isolates showed mutations at codons N51**I** and S108**N** (Table 2). A large proportion of the isolates (54%) carried the mutant genotype (**I**C**N**I), while 50 (46%) isolates harboured the wild-type (NCSI) (Table 2).

#### *dhps*

Unlike the other drug-resistant genes, no mutation was detected at all codons 436, 437, 540 and 613, which are implicated in sulphadoxine resistance.

## Discussion

Many sites in the Arabian Peninsula turned into “malaria-free” following intensive control efforts mounted over a relatively short period of time [1]. In contrast, in Yemen and southwest Saudi Arabia malaria remains resilient to control efforts. Some of the challenges facing the prospect of elimination, in these sites, is the introduction of drug resistance parasites via asymptomatic carriers. The present study examined the extent of genetic diversity and distribution of drug-resistant genes among of *P. falciparum* in some major foci in Yemen.

A high degree of diversity of non-drug-resistant genes (*msp-2* and *pfg377* and microsatellites in Chr 8) was seen among *P. falciparum* in Yemen. The *He* index ranged between 0.51 and 0.89, suggesting a high rate of transmission and a bigger parasite population size than anticipated in this region. Both allelic diversity and prevalence of mixed-genotype infection were high, 57% of the isolates harboured more than one allele at any of the examined loci, with a mean of 1.8 genotypes per infected individual. Assuming that each clone is readily transmissible to mosquito, the rate of cross-mating and subsequent recombination is expected to be high [25]. This agrees with the observed random association between loci and absence of geographical differentiation among *P. falciparum* in the three sites (Dhamar, Hodeidah and Taiz) (*F*_*st*_ < 0.05). Such a pattern of parasite structure is similar to that seen among *P. falciparum* populations in Africa [29,30]. These findings imply presence of a large effective population size (*Ne)* of *P. falciparum* in Yemen, as there is a direct relationship between the level of diversity and *Ne*[31-33]*.* This represents a challenge to control efforts in the whole region, as parasites can readily migrate from Yemen into other sites in the Arabian Peninsula, such as Oman [11], UAE [34] and Saudi Arabia [35], where transmission has been interrupted. In addition to local foci, imported malaria, via numerous migrations from Africa/Asia, can enrich diversity of parasites in the Arabian Peninsula. Over the past two decades political instability has resulted in mass movement of people from the Horn of Africa (Somalia, Eritrea and Ethiopia) into Yemen and Saudi Arabia [5]. Travellers from malaria-endemic areas can carry long-lasting, transmissible, asymptomatic parasitaemia [36,37]. Multiple introductions of distinct genetic sources could in part explain the high level of parasite diversity. However, the lack of genetic differentiation between parasites in different sites suggests that the prevailing epidemiological and demographical factors in Yemen are favorable to parasite dispersal. Imported malaria can, therefore, jeopardize the current success of control in the region despite the presence of a strong public health infrastructure. For example, countries such as Oman experience regular epidemics due to imported malaria [11]. To sustain malaria control and achieve elimination in the peninsula efforts should be directed to foci with on-going transmission, such as Yemen and Saudi Arabia.

The distribution of drug resistant genotypes seen in the present study fits with the history of anti-malarial usage, and possible migration of parasites from Africa and Asia into Yemen. The emergence of CQR in Yemen in 1989 Yemen [2,7,9,38,39], coincided with its appearance in close countries in East Africa [12,16,40] and Asia, Saudi Arabia [3], Iran and Pakistan [3,41-46]. The presence of two distinct *pfcrt* genotype, CV**IET** (89%) and **S**VMN**T** (4%) suggests that CQR in Yemen has evolved from at least two different origins. Globally, there are five genotypes of *pfcrt* CV**IET**, **S**VMN**T**, **S**V**IET**, CVMN**T** and CV**T**N**T**[47]. The CV**IET** genotype is predominant in Africa [48], while the **S**VMN**T** is common in Asian countries close to the Arabian Peninsula, such as Iran [43], India [49] and Pakistan [41,42]. This agrees with the hypothesis of frequent gene flow of African/Asian parasites into Yemen, and suggests that the CQR genotype (CV**IET**) is probably introduced via Africa, while genotype **S**VMN**T** has come from Asia. Analysis of microsatellites around *pfcrt* will allow further investigation to the origin of *pfcrt* resistance genotypes [29,48].

Similar to *pfcrt*, high prevalence of *pfmdr1* mutations was seen in Yemen. Mutations in codon 86**Y** has been linked to CQR, while those at codons 184, 1034, 1042 and 1246 are related to mefloquine (MQ), amodiaquine (AQ), halofantrine (HF) and quinine resistance [32,50,51]. In the present study, 86 (80%), 107 (99%) and 108 (100%) of the examined *P. falciparum* isolates carried alleles 86N and 184F and 1246D, respectively. Selection of *pfmdr1* 86N and 184F in recrudescent *P. falciparum* parasites following lumefantrine suggested a possible role of these alleles in the development of tolerance/resistance to lumefantrine [37,52]. Increased prevalence of *pfmdr1* 86N, 184F and 1246D haplotype was seen in Zanzibar after several years of extensive use of artesunate-amodiaquine (ASAQ) [53]. A recent study has confirmed that *P. falciparum* parasites with the *pfmdr1* N86/184F/D1246 haplotype can withstand 15-fold higher lumefantrine blood concentrations compared to those with the 86Y/Y184/1246Y haplotype [54]. The relatively high prevalence of the wild-type allele 86 N (80%) in the present study cannot be explained by withdrawal of CQ in Yemen, as the drug is still in use [55], and *pfcrt*76**T** remains at a prevalence of 89%. It is therefore, more likely that the pattern of *pfmdr1* polymorphisms, seen in Yemen, is due to resurgence in use of artesunate-SP combination. In addition to codon 86 and 184, mutations at the codons 1034 and 1042 were also high in Yemen; nonetheless no mutation was seen at codon 1246. Further longitudinal surveillance of *pfmdr1* mutations, coupled with *in vivo* testing, should be considered to examine the impact of these polymorphisms on efficacy of ACT in Yemen.

With regard to genes controlling *P. falciparum* response to SP, a previous study has detected the mutant allele dhfr-59**R** among 4 out of 99 isolates in Lahj governorate, southeast of Yemen, and no information was given on other codons 51, 108 and 164 [10]. However, the present study revealed high prevalence of mutations at codons 51 and 108 of the *dhfr*, while no mutation was seen at codon 59. This inconsistency can be attributed to the probability that some of the samples examined by Mubjer et al. [10] may have been collected from expatriates. If mutation 59R exists among local parasites in Yemen, it would have increase in frequency following recent shift to us of artesunate + sulphadoxine-pyrimethamine (SP) as first line therapy [55]. Epidemiological evidence suggests that *dhfr* mutations starts at codon 108, yielding low level resistance to pyrimethamine [38], then the resistance increases with acquisition of extra mutations at codons 51 or 59 and 164 [56]. Analysis of evolution pattern of *dhfr* genotypes shows that the high resistance genotype (51**I**, 59**R**, 108 **N**) accumulates mutations in sequence. The most common orders of mutation are 108 **N**, 59**R**, 51**I** or 108 **N**, 51**I**, 59**R**[57]. These cumulative mutations in *dhfr* restore the parasite fitness and allow it to overcome the effect of the drug [58]. *dhfr* mutations appear earlier than *dhps* mutations in the development of resistance to SP combined effect [59], and *P. falciparum* has to gain mutations in *dhfr* and *dhps* genes to develop resistance to SP, thus absence of *dhps* mutation in Yemen suggest that the SP will be effective for some years to come. Nonetheless, the occurrence of high level resistance *dhfr* and *dhps* genotypes linked to SP failure in areas close to Yemen, such as Iran and Pakistan (100%), the Horn of Africa; Sudan (72.7%) and Ethiopia (84.5%) [60-64], necessitate the need for regular surveys to monitor possible emergence of these mutations into Yemen for timely change in drug policy.

## Abbreviations

ACT: Artemisinin combination therapy; SP: Sulphadoxine-pyrimethamine; Pfcrt: *P*. *falciparum* chloroquine transport; pfmdr1: *P*. *falciparum* multidrug resistant; dhfr: Dihydrofolate reductase gene; dhps: Dihydropteroate synthase gene; msp-2: Merozoite surface protein-2; pfg377: *P. falciparum* gametocyte antigen; PCoA: Principle coordinate analysis; He: Expected heterozygosity; Fst: Fixation index.

## Competing interests

The authors declare that they have no competing interests.

## Authors’ contributions

SA-H, carried out the laboratory work, acquired the data, performed analysis and contributed to preparation of the manuscript. ZA-H, HA-F, AB-P and HB contributed to study design, analysis and interpretation of data, supervised laboratory work and revised the manuscript for important intellectual content. AMA-M, MAKM, MI and HB contributed to concept development and study design, edited and revised the manuscript critically for content. All authors read and approved the final manuscript.

## Supplementary Material

Additional file 1 **Alleles of 3 microsatellites (MS) on chromosomes 8, *msp-2* and *pfg377 *genes among 108 *Plasmodium falciparum* isolates in Yemen.** Click here for file
