# Supplementary material for: Genetic diversity of Plasmodium falciparum and distribution of drug resistance haplotypes in Yemen
Source: Malar J. 2013 Jul 15;12:244. doi: 10.1186/1475-2875-12-244 (PMC3729657; doi:10.1186/1475-2875-12-244)
Supplement: Additional file 1 — Alleles of 3 microsatellites (MS) on chromosomes 8, msp-2 and pfg377 genes among 108 Plasmodium falciparum isolates in Yemen. [file 1475-2875-12-244-S1.docx]

**Additional file 1. Alleles of 3 microsatellites (MS) on chromosomes 8, *msp-2* and *pfg377* genes among 108 *Plasmodium falciparum* isolates in Yemen**

|  | Yemen | | | |
| --- | --- | --- | --- | --- |
|  | Alleles | Taiz | Dhamar | Hodeidah |
|  |  | n(%) | n(%) | n(%) |
| MS 4.3kb | 98 | 1 (1%) | 0(%) | 0(%) |
|  | 100 | 3(3%) | 1 (1%) | 1 (1%) |
|  | 103 | 19(17.6%) | 6(5.5%) | 2(2%) |
|  | 105 | 13(12%) | 11(10%) | 6(5.5%) |
|  | 107 | 4(4%) | 7(6.5%) | 2(2%) |
|  | 109 | 10(9%) | 2(2%) | 5(4.6%) |
|  | 111 | 6(5.5%) | 1 (1%) | 1 (1%) |
|  | 113 | 6(5.5%) | 1 (1%) | 0(%) |
| MS 7.7kb | 104 | 4(4%) | 4(4%) | 1 (1%) |
|  | 106 | 8(7.4%) | 5(4.6%) | 7(6.5%) |
|  | 108 | 2(2%) | 0(%) | 0(%) |
|  | 110 | 0(%) | 1 (1%) | 1 (1%) |
|  | 112 | 12(11%) | 2(2%) | 0(%) |
|  | 116 | 1 (1%) | 0(%) | 1 (1%) |
|  | 118 | 4(4%) | 1 (1%) | 1 (1%) |
|  | 120 | 15(13.9%) | 5(4.6%) | 3(3%) |
|  | 122 | 7(6.5%) | 7(6.5%) | 3(3%) |
|  | 124 | 7(6.5%) | 0(%) | 0(%) |
|  | 126 | 1 (1%) | 2(2%) | 0(%) |
|  | 128 | 0(%) | 1 (1%) | 0(%) |
|  | 130 | 1 (1%) | 1 (1%) | 0(%) |
| MS 0.8kb | 109 | 1 (1%) | 0(%) | 0(%) |
|  | 113 | 1 (1%) | 0(%) | 0(%) |
|  | 115 | 34(31.5%) | 0(%) | 0(%) |
|  | 117 | 1 (1%) | 1 (1%) | 0(%) |
|  | 119 | 0(%) | 1 (1%) | 2(2%) |
|  | 121 | 3(3%) | 1 (1%) | 2(2%) |
|  | 123 | 3(3%) | 0(%) | 0(%) |
|  | 125 | 12(11%) | 3(3%) | 1 (1%) |
|  | 128 | 2(2%) | 15(13.9%) | 8(7.4%) |
|  | 131 | 1 (1%) | 2(2%) | 1 (1%) |
|  | 134 | 1 (1%) | 5(4.6%) | 2(2%) |
|  | 137 | 0(%) | 1 (1%) | 1 (1%) |
| *msp-2* | 3D7 | 20(18.5%) | 13(12%) | 10(9%) |
|  | FC27 | 42(38.9%) | 16(14.8%) | 7(6.5%) |
| *pfg377* | 269 | 2(2%) | 0(%) | 0(%) |
|  | 290 | 3(3%) | 2(2%) | 1 (1%) |
|  | 311 | 18(16.7%( | 10(9%) | 4(4%) |
|  | 332 | 31(28.7%) | 15(13.9%) | 7(6.5%) |
|  | 353 | 7(6.5%) | 2(2%) | 5(4.6%) |
